# Effect of Black Soybean Koji Extract on Glucose Utilization and Adipocyte Differentiation in 3T3-L1 Cells

**DOI:** 10.3390/ijms15058280

**Published:** 2014-05-09

**Authors:** Chi-Chang Huang, Wen-Ching Huang, Chien-Wen Hou, Yu-Wei Chi, Hui-Yu Huang

**Affiliations:** 1 Graduate Institute of Sports Science, National Taiwan Sport University, Taoyuan 33301, Taiwan; E-Mail: d301090007@gmail.com; 2 Graduate Institute of Athletics and Coaching Science, National Taiwan Sport University, Taoyuan 33301, Taiwan; E-Mail: magicpica521@gmail.com; 3 Department of Sports Sciences, University of Taipei, Taipei 11153, Taiwan; E-Mail: om65726@yahoo.com.tw; 4 Department of Food Science, Nutrition, and Nutraceutical Biotechnology, Shih Chien University, Taipei 10462, Taiwan; E-Mail: showmyself74@hotmail.com

**Keywords:** black soybean koji, 3T3-L1 preadipocytes, insulin, adipokines

## Abstract

Adipocyte differentiation and the extent of subsequent fat accumulation are closely related to the occurrence and progression of diseases such as insulin resistance and obesity. Black soybean koji (BSK) is produced by the fermentation of black soybean with *Aspergilllus awamori*. Previous study indicated that BSK extract has antioxidative and multifunctional bioactivities, however, the role of BSK in the regulation of energy metabolism is still unclear. We aimed to investigate the effect of glucose utilization on insulin-resistant 3T3-L1 preadipocytes and adipogenesis-related protein expression in differentiated adipocytes with BSK treatment. Cytoxicity assay revealed that BSK did not adversely affect cell viability at levels up to 200 μg/mL. The potential for glucose utilization was increased by increased glucose transporter 1 (GLUT1), GLUT4 and protein kinase B (AKT) protein expression in insulin-resistant 3T3-L1 cells in response to BSK treatment. Simultaneously, BSK inhibited lipid droplet accumulation in differentiated 3T3-L1 cells. The inhibitory effect of adipogenesis was associated with downregulated peroxisome proliferator-activated receptor γ (PPARγ) level and upregulated Acrp30 protein expression. Our results suggest that BSK extract could improve glucose uptake by modulating GLUT1 and GLUT4 expression in a 3T3-L1 insulin-resistance cell model. In addition, BSK suppressed differentiation and lipid accumulation in mature 3T3-L1 adipocytes, which may suggest its potential for food supplementation to prevent obesity and related metabolic abnormalities.

## 1. Introduction

Black soybean contains high-quality proteins and isoflavones. The seed coats contain anthocyanin, so they are darker than those of other soybeans [1]. In the traditional Taiwanese food fermentation industry, black soybean koji (BSK) mould has been widely used as the starter for preparing BSK, such as In-yu black sauce and In-si, the dried by-product of black soybean sauce. Solid black soybean has been fermented with generally recognized as safe (GRAS) filamentous fungi including *Aspergillus awamori*, *A. oryzae* (BCRC 30222), *A. sojae* (BCRC 30103), *Rhizopus azygosporus* (BCRC 31158) and *Rhizopus* sp. (No. 2) [2]. Black soybean fermented to BSK undergoes biotransformation that enhances its biological contents, such as aglycone isoflavone including daidzein, glycitein and genistein, which have several useful biological properties including antioxidant activity, antimutagenic activity, improvement in menopausal syndrome, and reduced risk of atherosclerosis [2–5].

Obesity increases the risk of cardiovascular disease in adults and has been strongly associated with insulin resistance in hyperglycemic people and those with type 2 diabetes [6]. With a 10% to 15% weight loss maintained over time, obese adults have shown sustained improvement in cardiovascular risk [7]. The pathophysiology of the development of type 2 diabetes is complex and multifactorial. Obesity is believed to lead to insulin resistance and increased circulating insulin concentrations over time. Recently, two functional proteins have been found related to insulin stimulate glucose transport. Glucose transporter 1 (GLUT1) belongs to constitutive glucose transporter and GLUT4 has been considered an insulin-sensitive glucose transporter. In addition, peroxisome proliferator-activated receptor γ (PPARγ) and adiponectin (Acrp30) protein are key modulators of insulin sensitivity and glycemic homeostasis [8]. PPARγ, a member of the PPAR subfamily of nuclear hormone receptors, has been identified as a molecule of differentiation-dependent regulatory factors of adipogenesis and a specific enhancer of the adipocyte fatty acid-binding protein (aP2) gene [9]. Adiponectin is an adipose-specific secretory protein in the blood circulation, and the level of adiponectin is lower in obese than in lean subjects. The administration of adiponectin was shown to improve insulin resistance in animal models [10]. In addition, upregulation of adiponectin expression could increase insulin sensitivity of heterozygous PPARγ knockout mice. Therefore, adiponectin activation may provide an important therapeutic strategy for obesity-linked disorders e.g., type 2 diabetes and metabolic syndrome [11]. Their protein expression could mediate lowering of free fatty acid levels, enhance *in vivo* insulin sensitivity and lower glucose levels in rodents [12–14].

Obesity is associated with a chronic inflammation, an abnormal cytokine production and activation of inflammatory signaling pathways in adipose tissue. The relationship between obesity and insulin resistance is related to dysregulation of endocrines, inflammatory, neural, and cell-intrinsic pathways. Obesity-associated insulin resistance is considered as a major risk factor for type 2 diabetes [15]. Increased secretion of adipokines including leptin, Acrp30 and resistin from adipocytes can modulate insulin signaling and lead to insulin resistance [16]. Therefore, these adipokines with increased adiposity in obesity exacerbate insulin resistance.

No studies have investigated BSK extract in preventing obesity and related metabolic abnormalities. We thus studied whether BSK could inhibit adipogenesis of 3T3-L1 adipocytes in a cell model. In addition, we examined the possible mechanism to support potential BSK improvement in insulin-resistant obesity.

## 2. Results and Discussion

### 2.1. Analysis of Isoflavones in Black Soybean Koji (BSK)

The types of isoflavone concentrations in BSK fermented with *A. awamori* at 30 °C for 72 h are in Table 1. The predominant isoflavone components were the glucoside form of genistin and daidzin at 1533.21 and 986.5 μg/g, respectively. The isoflavone aglycones including daidzein and genistein were at 3253.93 and 2329.67 μg/g, respectively. Moreover, the concentrations of malonylgenistin and malonyldaidzin were 2329.67 and 2329.67 μg/g, respectively. Endogenous β-glucosidase in soybean is reported to be able to convert isoflavone glucosides into aglycones, in agreement with the increase in the amount of isoflavone aglycones and decrease in isoflavone glucosides during soybean soaking in water and processing [17]. Previous studies have demonstrated that isoflavones have anti-obesity or anti-adipogenesis function by suppressing obesity-related transcription through a feedback mechanism on adiponectin, adipoR1, adipoR2, and 5′ adenosine monophosphate-activated protein kinase (AMPK), or by targeting the PI3K/AKT signaling pathway [18,19]. Therefore, we suggest that isoflavone-rich BSK extract may also have similar functions against adipogenesis or insulin resistance.

### 2.2. BSK Induced Cytotoxic Effects in 3T3-L1 Preadipocytes

We used MTT assay with 3T3-L1 preadipocytes to study the toxicity of BSK extract at different concentrations (25, 50, 100 and 200 μg/mL). Cell viability with BSK-25, BSK-50, BSK-100 and BSK-200 was from 99.96 ± 0.08 to 100.85 ± 0.10 (Table 2), with no difference in concentrations as compared with vehicle treatment. Moreover, microscopy of monolayer integrity did not reveal cytotoxic effects (data not shown). BSK did not adversely affect cell viability at concentrations up to 200 μg/mL. This finding agrees with a previous study of lactate dehydrogenase release used to monitor the safety of other flavonoids in 3T3-L1 cells [20]. Cheng and collaborators reported that fermented black soybean by *Rhizopus oligosporous* exhibited selective cytotoxicity toward human hepatocellular carcinoma cells but did not affect normal human lung fibroblast cells [21]. In addition, fermented black soybean broth had minimal cytotoxic effects at <10 mg/mL in a normal human skin fibroblast cell line, Detroit 551 [22]. Therefore, BSK may be considered safe and non-cytotoxic if used at <200 μg/mL.

### 2.3. BSK Treatment Enhanced Glucose Utilization in 3T3-L1 Preadipocytes

The 3T3-L1 preadipocytes (7.2 × 10^5^ cells/mL) cultured in Dulbecco’s modified Eagle’s medium (DMEM)-high glucose medium were treated with indicated concentrations of BSK extract (25–200 μg/mL) for 60 h. Glucose utilization with BSK-25, BSK-50, BSK-100 and BSK-200 treatment in 3T3-L1 preadipocytes was from 0.03 to 0.11 × 10^−5^ mg/cell (Table 2). At 200 μg/mL, BSK conferred a 5.5-fold increase in glucose utilization as compared with vehicle treatment. Therefore, at 200 μg/mL, BSK could increase and promote glucose utilization.

### 2.4. BSK Induced GLUT1, GLUT4 and AKT Protein Expression in Insulin-Resistant 3T3-L1 Preadipocytes

**Figure 1.**
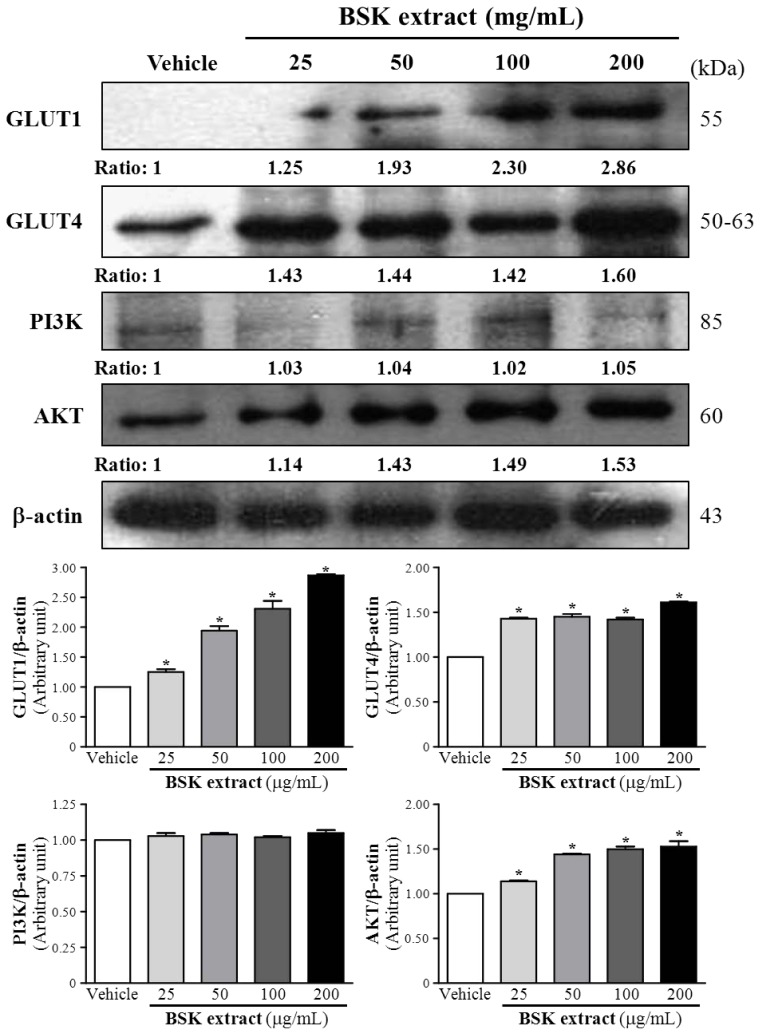
Western blot analysis of the effect of black soybean koji (BSK) extract on the protein expression of glucose transporter 1 (GLUT1), GLUT4, phosphatidylinositol-4,5-bisphosphate 3-kinase (PI3K) and protein kinase B (AKT) in 3T3-L1 preadipocytes. Preadipocytes were treated with vehicle (0.1% DMSO) or with different doses of BSK extract for 60 h. Data are mean ± SEM relative to vehicle treatment from 3 independent experiments. * *p* < 0.05 compared with vehicle.

GLUT1, GLUT4, PI3K and AKT are the key molecules involved in gluconeogenesis and energy metabolism. We treated 3T3-L1 preadipocytes, cultivated under differentiation conditions [23], with BSK extracts of different concentrations for 60 h to analyze the expression of GLUT1, GLUT4, PI3K, and AKT. GLUT1, GLUT4 and AKT expression was increased with BSK-25, BSK-50, BSK-100 and BSK-200 treatment as compared with vehicle treatment (Figure 1). However, PI3K expression did not differ from that with vehicle treatment. BSK, by upregulating GLUT1, GLUT4 and AKT expression but not PI3K expression, modulated gluconeogenesis and energy metabolism in 3T3-L1 preadipocytes. In adipocytes and skeletal muscle, insulin-stimulated glucose uptake is mediated by translocation of GLUT1 and GLUT4 to the plasma membrane from intracellular storage vesicles [24]. We found that BSK promoted glucose utilization in 3T3-L1 preadipocytes directly by elevating GLUT1 and GLUT4 expression.

### 2.5. BSK Inhibits Adipogenesis of 3T3-L1 Adipocytes

**Figure 2.**
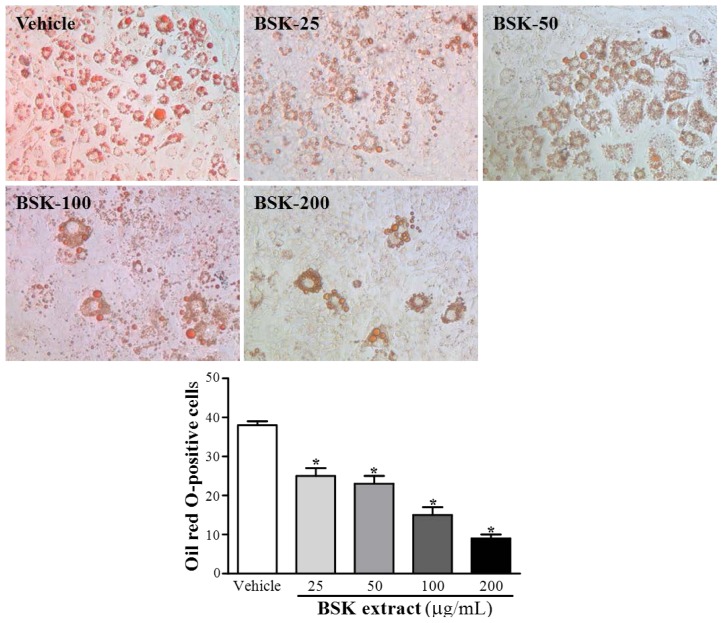
Effect of BSK extract on 3T3-L1 differentiated preadipocytes. During differentiation, cells were treated with vehicle (0.1% DMSO) or with the indicated concentrations of BSK extracts at 25, 50, 100 and 200 μg/mL, which were respectively designated the BSK-25, BSK-50, BSK-100 and BSK-200 groups. On day 8, cells underwent Oil-red O staining. Oil red O-positive cells calculated by the number of positive (red) cells. Data are mean ± SEM of 3 independent experiments. * *p* < 0.05 compared with vehicle.

3T3-L1 preadipocytes exposed to differentiation condition medium showed accumulation of a large amount of lipid in cytoplasm (Figure 2). We co-exposed preadipocytes to differentiation cocktail and different concentrations of BSK to test the ability to modulate 3T3-L1 cell differentiation. BSK treatment dose-dependently inhibited adipogenesis of 3T3-L1 cells as determined by the lipid droplet accumulation in cytoplasm (Figure 2). Lipid accumulation was substantially reduced with BSK-100 and BSK-200, with no difference between BSK-25, BSK-50 and vehicle groups.

**Figure 3.**
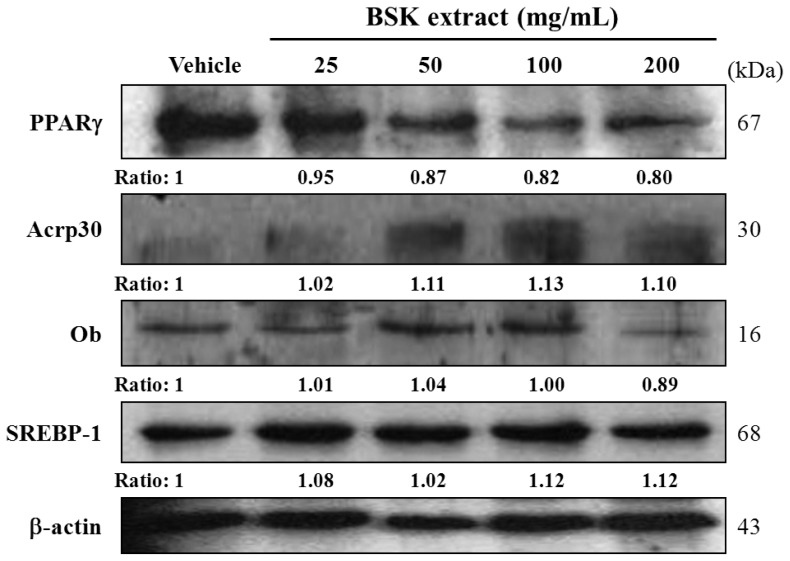

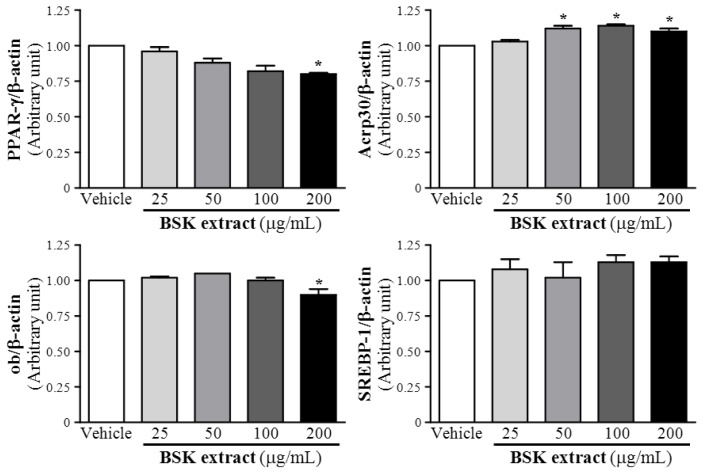
Western blot analysis of the effect of BSK extract on protein expression of PPARγ, Acrp30, Ob and SREBP-1 on day 8 in 3T3-L1 preadipocytes. During differentiation, cells were treated with vehicle (0.1% DMSO) or with BSK extract for 8 days at the indicated concentrations (25–200 μg/mL). Data are mean ± SEM relative to that of vehicle treatment from 3 independent experiments. * *p* < 0.05 compared with vehicle.

The 3T3-L1 preadipocytes could be induced to differentiate into adipogenic cell types with adipogenic stimulus. Transcription factors CCAAT enhancer-binding proteins (C/EBPs) and PPARγ play crucial roles required for adipocyte differentiation [25]. Our data showed that BSK extract suppressed PPARγ expression (Figure 3). Therefore, the lipid metabolism and accumulation could be inhibited in BSK-treated cells.

### 2.6. BSK Regulates Acrp30 and PPARγ Protein Expression in 3T3-L1 Adipocytes

3T3-L1 preadipocytes were treated with concentrations of BSK extract during differentiation for 8 days and PPARγ, Acrp30, Ob, and SREBP-1 protein levels were evaluated for possible effects on differentiation. Decreased expression and/or secretion of adiponectin (Acrp30, an adipocyte-specific hormone) is strongly associated with insulin resistance [26]. Thus, we examined the ability of BSK to modulate Acrp30 expression in mature 3T3-L1 adipocytes. Acrp30 protein level was increased with increased BSK concentration as compared with vehicle treatment (Figure 3). The dose from 50 to 200 μg/mL could significantly upregulate Acrp30 expression. However, PPARγ and Ob protein levels were significantly decreased with BSK-200 treatment, by 20% and 11%, respectively, as compared with vehicle treatment. SREBP-1 protein level did not differ with treatment. Therefore, BSK increased the expression of Acrp30 and promoted energy metabolism of adipocytes at as low as 50 μg/mL concentration. PPARγ is a ligand-activated transcription factor that regulates adipogenesis and is expressed during the early to middle stages of adipocyte differentiation [27]. Previous study reported that black soybean anthocyanin-treated cells expressed considerably lower levels of PPARγ as compared with vehicle treatment [28]. We found that 200 μg/mL of BSK extract could significantly reduce the expression level of PPARγ, which agreed with previous reports of the inhibition of adipocyte differentiation and diminished lipid accumulation. Here, we found that BSK could attenuate lipid accumulation in differentiating adipocytes by regulating Acrp30 and PPARγ protein expression.

The sterol regulatory element binding protein (SREBP) has been shown to regulate several cholesterol synthesis-associated genes including HMG-CoA reductase and reductase, and fatty acid synthesis-related genes such as acetyl CoA carboxylase (ACC), fatty acid synthase (FAS) and glycerol-3-phosphate acyltransferase [29]. Acrp30/adiponectin is secreted by adipose tissues and is downregulated in obesity-linked insulin resistance. Ob/leptin is synthesized and secreted by adipocytes and acts primarily to regulate energy homoeostasis [30]. Obesity is associated with leptin resistance and increased circulating leptin levels. In this study, BSK extract increased levels of adiponectin and decreased levels of leptin and PPARγ (Figure 3) that could contribute to attenuate lipid accumulation during adipogenesis (Figure 2) and ameliorate the insulin resistance via glucose utilization evaluation (Table 2).

## 3. Materials and Methods

### 3.1. BSK Extract Preparation

Black soybean (Tainan No. 3) was obtained from a local company (Shiuejia Farmers’ Cooperative, Tainan, Taiwan). BSK was prepared as described [2]. The lyophilized BSK was ground into powder (250 g) and extracted by 95% ethanol with sonication at 25 °C for 24 h. The extract was filtered through Whatman No. 1 filter paper. After filtration, the solvent was concentrated by use of a rotary evaporator freeze dryer (Büchi R-215, Westbury, NY, USA) to obtain the BSK ethanol extract (18.32 g).

### 3.2. Analysis of Isoflavones in BSK

BSK was extracted by use of high-performance liquid chromatography (HPLC)-grade methanol containing 5000 ppm benzoic acid as an internal standard, with sonication for 1 h and centrifuged at 10,000 rpm for 5 min. The supernatant of BSK extract was filtered and diluted 20 times to a final 0.6% containing 250 ppm internal standard. A portion of the sample was filtered through a 0.45-mm polytetrafluoroethylene filter unit (Alltech Associates, Deerfield, IL, USA) and analyzed by HPLC with an Atlantis dC18 column (5 μm, 4.6 × 250 mm). A linear HPLC gradient was composed of 0.1% (*v/v*) glacial acetic acid in water and 0.1% glacial acetic acid in acetonitrile. After the injection of 20 μL sample, 0.1% glacial acetic acid in acetonitrile was increased from 15% to 20% at 0–20 min, then increased to 24% within 10 min, maintained at 24% with 6 min, increased from 24% to 35% within 8 min, maintained at 35% with 6 min, decreased from 35% to 15% within 6 min and maintained to 15% at the end of 58-min duration. The individual isoflavone content was calculated as follows: isoflavone content (μg/g) = (AS/AIS) × (CIS/RRF) × (V/MS); where AS is peak area of isoflavone compound; AIS, peak area of internal standard; CIS, concentration of internal standard; V, total volume of extract solution; MS, sample weight; RRF, relative response factor.

### 3.3. Cell Culture and Insulin-Resistant Adipocyte Induction

Murine 3T3-L1 cells, purchased from the Bioresource Collection and Research Center (BCRC, Hsinchu, Taiwan), were grown in Dulbecco’s modified Eagle’s medium (DMEM) with 1% antibiotics (PEN-STREP-AMPHO), 1 mM sodium pyruvate, 4.5 g/L glucose and 10% fetal bovine serum under 37 °C, 5% CO_2_ and 95% humidity until confluent. Differentiation was induced by changing the medium to DMEM supplemented with 0.5 mM 3-isobutyl-1-methylxanthine, 1 mM dexamethasone, 10% fetal bovine serum, and 10 mg/L insulin for the next 2 days, then differentiation medium was replaced with previous maintenance medium and medium was changed every 48 h until about 8 days. The differential process was as described [31] with modification.

### 3.4. 3-(4,5-Dimethylthiazol-2-yl)-2,5-diphenyltetrazolium Bromide Assay (MTT) Assay

3T3-L1 cells were maintained and cultured until 80% confluence, and cells were trypsinized with 1× Trypsin-EDTA (Sigma Chemical, St. Louis, MO, USA), then counted by trypan blue dye exclusion and seeded 1 × 10^4^ into 96-well plates (100 μL media/well) for 24 h with cellular complete attachment. The BSK extract stock, dissolved in DMSO, was diluted with medium for the indicated concentrations (25–200 μg/mL) and added into individual wells with 3 replications for 24-h incubation. Then, the sample medium was removed and washed with phosphate buffered saline (PBS) twice. The 100 μL medium containing MTT [3-(4,5-dimethylthiazol-2-yl)-2,5-diphenyltetrazolium bromide] at 0.5 mg/mL was added into wells for 2-h incubation. The unreactive supernatant was discarded and 200 μL DMSO was added for shaking for 10 min until the crystal was dissolved. The absorbance at 570 nm was measured with a full-length ELISA reader (Synergy2, Biotek, Winooski, VT, USA). The percentage of viable cells was calculated by setting cell viability without treatment to 100%.

### 3.5. Benedict Test to Detect Reducing Sugars

Benedict’s solution is designed to detect the presence of reducing sugars. In hot alkaline solutions, reducing sugars reduce the blue copper (II) ions to copper (I) oxide precipitate. After the reaction process, the color of the reaction mixture developed progressively from blue to green, yellow, orange and red. When conditions are carefully controlled, the color changes and the amount of precipitate formed depends on the amount of reducing sugars present, thereby providing an estimate of the concentration of glucose-equivalent reducing sugars present in a sample.

The test samples, 90 μL, were added into 1 mL benedict reagent by mixing and reaction at 100 °C for 5 min. The reaction samples were centrifuged for 5 min and 200 μL was obtained for spectrophotometry at 420 nm. The concentrations of the medium of test samples were calibrated with standard glucose solutions. The glucose uptake of treated cells was calculated as the difference within 24 h and divided by cell numbers to represent glucose utilization.

### 3.6. Western Blot Analysis

Primary antibodies against anti-oxidation associated proteins, including PPARγ, Acrp30, Ob, SREBP-1, GLUT4, GLUT1, and AKT, were used for western blot analysis. The other antibodies including β-actin as and internal control and secondary antibodies were from Santa Cruz Biotechnology (Santa Cruz, CA, USA). The detailed procedures of western blot analysis were as previously described [32].

### 3.7. Oil-Red O Staining

The differentiated 3T3-L1 adipocytes with indicated BSK extract treatments were washed twice with PBS and fixed with 3.7% formaldehyde for 1 h. After removal of the fixing solution, cells were stained with filtered Oil-red O solution (60% isopropanol and 40% water) for 15 min, then staining solution was removed, and plates were rinsed with water and dried. The stained lipid droplets were viewed as red under a light microscope equipped with a charge-coupled device (CCD) camera (BX-51, Olympus, Tokyo, Japan). The Oil red O-positive cells were measured by use of AxioVision software (Carl Zeiss MicroImaging, Inc., Thornwood, NY, USA).

### 3.8. Statistical Analysis

Data are shown as mean ± SEM. To evaluate differences among the groups, data were analyzed by one-way analysis of variance (one-way ANOVA) with use of SAS 9.0 (SAS Inst., Cary, NC, USA). *p* < 0.05 was considered statistically significant.

## 4. Conclusions

Fermented black soybean extract may provide bioactivity to regulate adipogenesis and may be used in health food applications for adipose regulation. In this study, BSK extract showed anti-adipogenic function via regulation of PPARγ and adiponectin expression. BSK extract also ameliorated obesity-associated insulin resistance and increased glucose utilization by upregulation of GLUT protein expression. Therefore, we suggest that BSK may be an effective agent for treating obesity induced insulin resistance and that the active compounds in BSK could be further investigated for the detailed molecular mechanisms involved in adipogenesis regulation.

## Figures and Tables

**Table 1.** Isoflavone contents of black soybean koji (BSK).

Isoflavone	Area (%)	Area sample/area IS	Concentration (ppm)	Isoflavone content (μg/g)	RRF (BA)
Benzoic acid	18.77	1.00	5000.00	-	1
Daidzin	3.41	0.18	118.38	986.50	7.67
Genistin	5.57	0.30	183.99	1533.21	8.06
Malonyldaidzin	6.61	0.35	301.55	2512.94	5.84
Malonylgenistin	3.43	0.18	103.96	866.34	8.78
Daidzein	14.61	0.78	390.47	3253.93	9.97
Genistein	12.81	0.68	279.56	2329.67	12.21
Total isoflavone	-	-	-	11,482.60	-

RRF, relative response factor; IS, internal standard; BA, benzoic acid.

**Table 2.** Effects of BSK Extract on Cytotoxicity and Glucose utilization in 3T3-L1 Preadipocytes.

Concentration (mg/mL)	Cell viability (%)	Glucose utilization (mg/cell)
vehicle (0.1% DMSO)	100.0 ± 4.7	0.02 × 10^−5^
25	99.4 ± 4.5	0.03 × 10^−5^
50	101.3 ± 6.4	0.04 × 10^−5^
100	99.1 ± 5.6	0.03 × 10^−5^
200	100.2 ± 3.9	0.11 × 10^−5^ *

Data are mean ± SEM relative to that of vehicle (*n* = 3).

* , *p* < 0.05 compared with vehicle;

DMSO, dimethyl sulfoxide.
